# Detecting Flood‐Rich and Flood‐Poor Periods in Annual Peak Discharges Across Europe

**DOI:** 10.1029/2019WR026575

**Published:** 2020-07-09

**Authors:** David Lun, Svenja Fischer, Alberto Viglione, Günter Blöschl

**Affiliations:** ^1^ Institute of Hydraulic Engineering and Water Resources Management Vienna University of Technology Vienna Austria; ^2^ Institute of Hydrology, Water Resources Management and Environmental Engineering Ruhr‐University Bochum Bochum Germany; ^3^ Department of Environment, Land and Infrastructure Engineering Politecnico di Torino Turin Italy

**Keywords:** Flood changes, Flood frequency, Scan statistics

## Abstract

This paper proposes a method from Scan statistics for identifying flood‐rich and flood‐poor periods (i.e., anomalies) in flood discharge records. Exceedances of quantiles with 2‐, 5‐, and 10‐year return periods are used to identify periods with unusually many (or few) threshold exceedances with respect to the reference condition of independent and identically distributed random variables. For the case of flood‐rich periods, multiple window lengths are used in the identification process. The method is applied to 2,201 annual flood peak series in Europe between 1960 and 2010. Results indicate evidence for the existence of flood‐rich and flood‐poor periods, as about 2 to 3 times more anomalies are detected than what would be expected by chance. The frequency of the anomalies tends to decrease with an increasing threshold return period which is consistent with previous studies, but this may be partly related to the method and the record length of about 50 years. In the northwest of Europe, the frequency of stations with flood‐rich periods tends to increase over time and the frequency of stations with flood‐poor periods tends to decrease. In the east and south of Europe, the opposite is the case. There appears to exist a turning point around 1970 when the frequencies of anomalies start to change most clearly. This turning point occurs at the same time as a turning point of the North Atlantic Oscillation index. The method is also suitable for peak‐over‐threshold series and can be generalized to higher dimensions, such as space and space‐time.

## Key Points

- A method from Scan statistics is proposed for identifying flood‐rich and flood‐poor periods in flood discharge records
- There is evidence of flood‐rich and flood‐poor periods based on analyzing 2,201 flood series in Europe between 1960 and 2010
- In the northwest of Europe, the frequency of flood‐rich periods tends to increase over time, particularly for large return periods

## 1 Introduction

Recent studies indicate that flood discharge regimes and associated probabilities have changed in recent decades in some parts of the world (Hall et al., 2014; Hodgkins et al., 2017; Blöschl, Hall, et al., 2019). Changing flood probabilities have implications for estimating design values of flood infrastructure, flood risk zoning and insurance (Khare et al., 2015). Trend analysis remains the reference procedure for detecting changes in systematic flood discharge series, while historical data are often analyzed in terms of periods with unusually few or many floods (Hall et al., 2014). However, it has been suggested that changes may be more complex than simple trends, with the identification of flood‐rich and flood‐poor periods therefore a more suitable approach for detecting flood changes from systematic data. Trend models usually assume a constant change with time of the mean or the parameters of the flood distribution. Previous studies suggest that this assumption does not always apply (Hall et al., 2014). Modeling changes as a different kind of inhomogeneity in time, that is, periods with unusually many high (or low) discharge observations allows for more flexibility in the nature of change if the existence, the beginning and the end of these periods are inferred from the data. The existence of floodrich periods refers to Unsolved Problem Number 9 of Blöschl, Bierkens, et al. (2019) Additionally, the presence of flood‐rich and flood‐poor periods, implying temporal clustering, may make statistical estimation procedures more difficult, as additional parameters and assumptions are needed (Khare, et al., 2015 Serinaldi & Kilsby, 2015). This clustering is usually defined as a statistically significant deviation from a time‐homogeneous Poisson process in the context of peak‐over‐threshold (POT) data, where threshold exceedances are treated as events.

A number of studies have identified the presence of clustering, or flood‐rich/flood‐poor periods, in historical flood data. Swierczynski et al. (2013) reconstructed 7,100‐year series of flood events from sediment records of a lake in the Eastern Alps and report decadal and multidecadal clustering with flood‐rich periods of 30‐ to 50‐year duration. Schmocker‐Fackel and Naef (2010) identified periods with unusually frequent flood events lasting between 30 and 100 years in 14 historical records of up to 500‐year lengths in Switzerland. They suggested these periods to be in phase with those in the Czech Republic, Spain and Italy. Jacobeit et al. (2003) found associations between variations in flood occurrence and large‐scale circulation modes for several central European catchments in the past 500 years. Similarly, Glaser et al. (2010) reported several periods with increased flood frequency of central European and Mediterranean rivers and coherence of these periods among the series.

Studies on interannual clustering based on systematic flood data are rarer (see Table 1 of Liu & Zhang, 2017). Analyzing 103 flood records of up to about 100‐year length, Mediero et al. (2015) found clustering in the Atlantic and continental regions of Europe but no clustering in the rest of the continent. Liu and Zhang (2017) found significant clustering in Southeastern Australia but little clustering in the rest of the continent. Merz et al. (2016) found significant intra‐annual clustering in most of the 68 catchments examined in Germany, while interannual clustering was only present in a few series. They longest series they examined exhibited interannual clustering. Both Merz et al. (2016) and Liu and Zhang (2017) reported that the number of series exhibiting significant clustering decreased with increasing return period.

Merz et al. (2016), Liu and Zhang (2017) and most other authors used POT flood series rather than annual maximum (AM) peak discharges, and their method for identifying clustering is tailored to POT series. In most countries, available AM series are longer than POT series, which would allow the use of more comprehensive data sets. In this paper we propose a method from Scan statistics that is suitable for identifying clustering in both AM series and POT series of floods, and it can also be used for other time series such as precipitation. The method yields a classical assessment of the compatibility of the most unusual cluster with the proposed statistical model in the form of a *p* value, as well as the position of the unusual period within the time series. By defining flood events as threshold exceedances, unusual periods refer to periods in the observations that are inconsistent with the reference condition of independent and identically distributed random variables (iid), by encompassing unusually many (or unusually few) events. These periods will be referred to as flood‐rich (or flood‐poor) periods. We apply the method to 2,201 annual series from 33 European countries for the period 1960 to 2010. The main questions addressed are the following: (1) Do flood‐rich and flood‐poor periods exist in Europe in the past five decades? (2) If they existed, did they exhibit spatiotemporal patterns?

## 2 Method

### 2.1 Overall Approach

A common assumption in flood frequency analysis is that observed flood discharge peaks can be modeled by a family of iid random variables. Given a set of observations of an iid‐process and a corresponding fixed quantile threshold *x*_*p*_, the process of an iid random variable exceeding the threshold can be modeled by a Bernoulli process, that is, a series of independent Bernoulli trials with success probability 1‐*p*, which can be used for checking the validity of the reference condition of an iid‐process.

In this paper, the clustering of flood events is investigated by testing the implications of the iid assumption on the arrangement of threshold exceedances: Given a time series of *n* observations and a window length *m*, the number of quantile exceedances in each window of *m* consecutive observations is recorded. The window with the highest number of recorded events is reported, as it is the most likely candidate for a flood‐rich period. Note that, although individual observations are assumed to be independent, the windows are not as they overlap in time. The corresponding probability, that is, how likely it is to encounter a window of consecutive observations of length *m* in which the given quantile *x*_*p*_ is exceeded at least *k* times, will be denoted as *P*(*k* ∣ *m*,*n*,*p*_*exceedance*_).

### 2.2 Scan Statistics

The probability *P*(*k* ∣ *m*,*n*,*p*_*exceedance*_) arises in different contexts in the literature, most prominently in the context of Scan statistics. Following Glaz et al. (2001), let *X*_1_,…,*X*_*n*_ be a time discrete sequence of integer‐valued random variables. Then the one‐dimensional unconditional discrete Scan statistic is defined as

(1) $$S_{m}=\max_{1\leq t\leq n-m+1}Y_{t}\;\text{with}\;Y_{t}=\sum_{i=t}^{t+m-1}X_{i}$$

*S*_*m*_ is the maximum number of observed counts in a series of overlapping sliding windows (of length m). The maximum is taken over the index *t*, which marks the beginning of the individual windows. Equation 1 applies to annual series, as used in this paper. For time‐continuous (POT) series, equation 1 would have to be modified (Glaz et al., 2001, p. 185; Wu et al., 2013). Let *X*_*t*_ be a sequence of Bernoulli trials *X*_*t*_~*B*(*p*). The probability of obtaining at least *k* successes within a window of length *m* in *n* realizations of *X*_*t*_, *P*(*S*_*m*_ ≥ *k*), is denoted *P*(*k* ∣ *m*,*n*,*p*). This probability can be used to test for the presence of clusters with respect to the reference condition of a time‐constant Bernoulli process.

Exact results for the distribution of the Scan statistic *S*_*m*_ are available (see Glaz et al., 2001 for an overview), as well as for the time‐continuous counterparts for some cases.

The distribution of the Scan statistic is connected to a statistical hypothesis test. The usual hypothesis in this context is time‐constant intensity of events or successes against a pulse‐alternative. In the case of the Bernoulli process the hypotheses are

(2) $$H_{0}:X_{i}\sim B\left(p_{0}\right)\;i=1,\ldots ,n$$

$$H_{1}:X_{i}\sim B\left(p_{0}\right)\;i=1,\ldots ,t-1,t+m,\ldots ,n\;\text{and}$$

$$X_{i}\sim B\left(p_{1}\right)\;i=t,\ldots t+m-1$$

The alternative hypothesis $H_{1}$ refers to subsections of the data with implausibly many (or few) events (under the null hypothesis), that is, flood‐rich or flood‐poor periods. The corresponding hypothesis test is a generalized likelihood ratio test (Fu & Curnow, 1990; Glaz & Naus, 1991). See Naus (1966) for the time‐continuous case.

(3) $$\Lambda \left(x\right)=\frac{L\left(p_{1}|x\right)}{L\left(p_{0}|x\right)}=\frac{\prod_{i=t}^{t+m-1}p_{1}^{x_{i}}{\left(1-p_{1}\right)}^{1-x_{i}}}{\prod_{i=t}^{t+m-1}p_{0}^{x_{i}}{\left(1-p_{0}\right)}^{1-x_{i}}}$$

The test rejects the null hypothesis for large values of the Scan statistic. Here it is assumed that *p*_0_ and the window size m are known, while the position of the window *t* and *p*_1_ are unknown, but *p*_1_ > *p*_0_. The Type I error rate *α* corresponds to the tail probability of the Scan statistic for fixed parameters *P*(*S*_*m*_ ≥ *k*) = *P*(*k* ∣ *m*,*n*,*p*) = *α*. $H_{0}$ is rejected if the maximum number of exceedances in a single window exceeds *k* for a defined significance level. For a given observed maximum number of exceedances in a single window equal to *k*, the *p* value of the corresponding test is *P*(*S*_*m*_ ≥ *k*), that is, the probability of observing *k* or more exceedances in a single window under the assumption of $H_{0}$.

We evaluated the distribution of the Scan statistic for Bernoulli trials, corresponding to the tail probabilities *P*(*k* ∣ *m*,*n*,*p*), using the approach of Fu (2001), by formulating the problem as a discrete‐time Markov chain. An advantage of this approach is that the distribution can be easily evaluated, assuming a change in the underlying distribution as specified in the alternative hypothesis of the generalized likelihood ratio test. This corresponds to the power of the test with respect to this alternative (Fu & Curnow, 1990 Wallenstein et al., 1994).

The variable *X*_*t*_ in equation 1 is now defined as binary, taking the value 1 if the flood peak *Q*_*t*_ is equal to or exceeds a threshold *Q*_*p*_ and 0 otherwise. However, the corresponding quantile *Q*_*p*_ is not known a priori. We used order statistics for estimating *Q*_*p*_ instead of other estimators such as L‐moments as order statistics allow for an exact test for the following reasons. Applying the Scan statistic to exceedances of a point estimate of a quantile derived from a finite sample via L‐Moments or similar estimators will result in a distortion of the level of the Scan statistic, as the estimate does not have the true prescribed exceedance probability. However, when the data‐generating process *X*_*t*_ is assumed to be real‐valued and iid, implying no ties are possible, the ranks of a finite sample are uniformly distributed over all possible permutations of the integers (1, …, *n*) (see, e.g., p. 206 of Hollander et al., 2013). This implies that every possible arrangement of the corresponding ranks is equally likely. By dichotomizing the data through exceedances of a quantile estimate obtained by order statistics, we know a priori how many exceedances will be observed and, under the null hypothesis, all possible arrangements of these exceedances over the *n* observations are equally likely. The associated clustering probabilities are given by the conditional discrete‐time Scan statistic (Glaz et al., 2001 Naus, 1974) where a number of trials is investigated, which can either result in a success (*X*_*t*_ = 1) or a failure *(X*_*t*_ = 0), but the total number of successes is known. While, in the unconditional case, the total number of successes is treated as a random variable *P*(*S*_*m*_ ≥ *k*) = *P*(*k* ∣ *m*,*n*,*p*), in the conditional case $P\left(S_{m}^{\prime }\geq k\right)=P\left(k|m,n,a\right)$, that is, instead of the individual success probability *p* the (known) total number of successes *a* is used. The distribution of the discrete‐time conditional Scan statistic under the hypothesis where all $\left(\begin{array}{c} n\\ a \end{array}\right)$ arrangements of successes are equally likely was estimated here via a Markov chain as well (Fu et al., 2012).

The quantile estimates ${\hat{Q}}_{p}$ via order statistics were obtained by the estimator of Makkonen and Pajari (2014):

(4) $${\hat{Q}}_{p}=\left(n+1\right)\left(Q_{\left(i+1\right)}-Q_{\left(i\right)}\right)\left(p-p_{i}\right)+Q_{\left(i\right)}\;\text{with}\;p_{i}=\frac{i}{n+1};\;p_{i}\leq p<p_{i+1}$$

where *Q*_(*i*)_ is an order statistic with associated nonexceedance probability *p*_*i*_. The number of years n of data is given for each series. The desired exceedance probabilities are fixed a priori, resulting in the number of observed events *a*. Therefore, the only free parameters are the window length *m* and the number of exceedances *k*. The 50%, 80%, and 90% quantiles were used, corresponding to 2‐, 5‐, and 10‐year flood thresholds. In this paper, any spatial correlations of the flood data were not accounted for as their effect was assumed to be small. Accounting for spatial correlations will likely reduce the number of anomalies and the magnitude of this effect should be examined in future studies.

**Figure 1:**
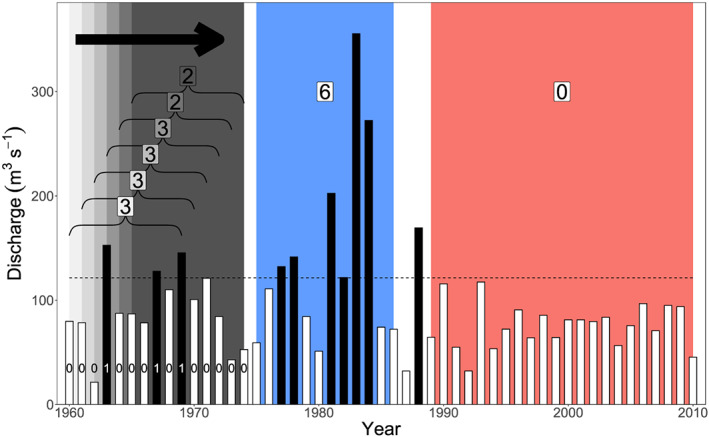
Illustration of discrete‐time conditional Scan statistic applied to discharge data: Sample of a GEV distribution. Dotted line indicates 80% quantile estimated via order statistics; exceedances are dark shaded bars and marked as 1 at the beginning. Number of exceedances in every window is shown for the first six windows. The blue area denotes a subset with an unusually high count of exceedances, whereas the red area denotes a subset with unusually few exceedances.

Figure 1 illustrates the application of this approach to a series of 51 simulated AM peak discharge observations sampled from a generalized extreme value distribution. The 80% quantile, corresponding to 5‐year events, is estimated via order statistics (equation 4), resulting, in this example, in 10 exceedances which are treated as events. Employing a 10‐year window, the number of exceedances of each set of 10 consecutive observations can be enumerated (first windows in Figure 1). The maximum is reached for 1975–1986 (three 10‐year windows all counting six exceedances). From Table 1 one can read the probability of observing at least six exceedances in any window for the given sample size and number of events, which can be interpreted as the corresponding *p* value. For a 10‐year window (blue window), the probability is rather small (0.0362). On the other hand, the probability of observing a period without any exceedances that is at least 22 years long is about 0.0172 for the given parameters under the null hypothesis (red window).

**Table 1:** *Distribution of the Scan Statistic (Tail Probability P) for a Sample Size n = 51, Number of Successes a = 10, Length of the Window m = 10, and Varying Maximum Number of Exceedances k*

*k*	1	2	3	4	5	6	7	8	9	10
*P*(*k* ∣ *m*,*n*,*a*)	1	1	0.99	0.79	0.24	0.04	2.68 10^−3^	9.19 10^−5^	1.18 10^−6^	3.29 10^−9^

*Note*. Probabilities are rounded to two digits.

The window length of a postulated flood‐rich anomaly is not known a priori. For flood‐rich periods, multiple windows are therefore used at the same time to check for a change in the underlying distribution. The test statistics and their *p* values were combined via the approach of Wu et al. (2013). They illustrate that a multiple window Scan statistics is generally advantageous in terms of power, when the pulse size under the alternative hypothesis is unknown; that is,

(5) $$1-P\left(S_{m_{j}}<k_{j},\text{for}\;\mathrm{all}\;j=1,\ldots ,w|n,a\right)$$

In equation 5 *w* windows of different lengths *m*_*j*_ are employed. Equation 5 is the complementary probability of having less than *k*_*j*_ exceedances in all windows for the respective window lengths *m*_*j*_. Window sizes of 5, 10, and 15 years are used. Based on those, as well as on the length of the individual series *n* and the number of events a derived from the prescribed event magnitudes, the number of exceedances k associated with a probability of less than 5% was derived. If that number of exceedances (or more) was actually detected in a series, that period was marked as significant. The observed number of events, derived via exceedances of order statistics, depends heavily on the threshold return period chosen. For instance, for the case of a 2‐year flood threshold, a series of 51 years results in 26 values that are at least as large as the quantile estimate derived from equation 4. Including a window of Size 5 in the combined probability (equation 5) in this case results in probabilities that are always bigger than 5%, which prevents significant finds at the 5% level. Therefore, only windows of size 10 and 15 were used for this case. Similarly, for 10‐year thresholds and shorter series only windows of Sizes 5 and 10 were investigated.

Flood‐poor periods were defined in a similar way, but no multiple window sizes were used. Given *n* observations and a number of events *a*, one can ask: What is the probability of an iid‐process producing a period of length *m* or longer without any events? By letting *m* vary, the value was chosen in a way that the probability of observing such a period was less than 5% under the null hypothesis. This means that flood‐poor periods were defined by the absence of events. Therefore, in contrast to flood‐rich periods, no multiple window sizes were used for flood‐poor periods.

## 3 Data

The detection procedure was applied to 2,370 AM peak discharge series for the period 1960 and 2010 from 33 European countries (Blöschl, Hall, et al., 2019). The selection of the time series followed the requirements for fitting a trend model in Blöschl, Hall, et al. (2019). For a series to be selected there had to be at least 40 years of observations between 1960 and 2010, with the record starting in 1968 or earlier, and ending in 2002 or later. In countries with especially high station density, such as Germany or Austria, only series with at least 49 years were used. In Cyprus, Italy and Turkey series only had to encompass 30 years to be accepted. For the Spanish series, the requirements with regard to the beginning and end were dropped because of the shorter records. These requirements are mainly for data continuity and completion between the beginning and end dates.

The detection of flood‐rich and flood‐poor periods with the proposed procedure requires a coherent set of observations. Out of the 2,370 selected series, 389 had one or more gaps. To increase the number of stations in the analysis, some of the gaps were filled. For series with two or fewer gaps of 2 years or shorter each, the missing observations were predicted by a simple linear regression model between the at‐site observations and the observations of a donor station. The donor station was selected as the station with the highest Pearson cross correlation of the flood peaks within a radius of 400 km. The resulting series were all visually inspected and checked for plausibility. In 220 series, a total of 302 missing observations was replaced. Out of these, 168, 37, and 18 exceeded the 2‐, 5‐, and 10‐year return period thresholds, respectively. The filled‐in values have very similar counts as the expected proportions, so there is very little bias due to filling in.

**Figure 2:**
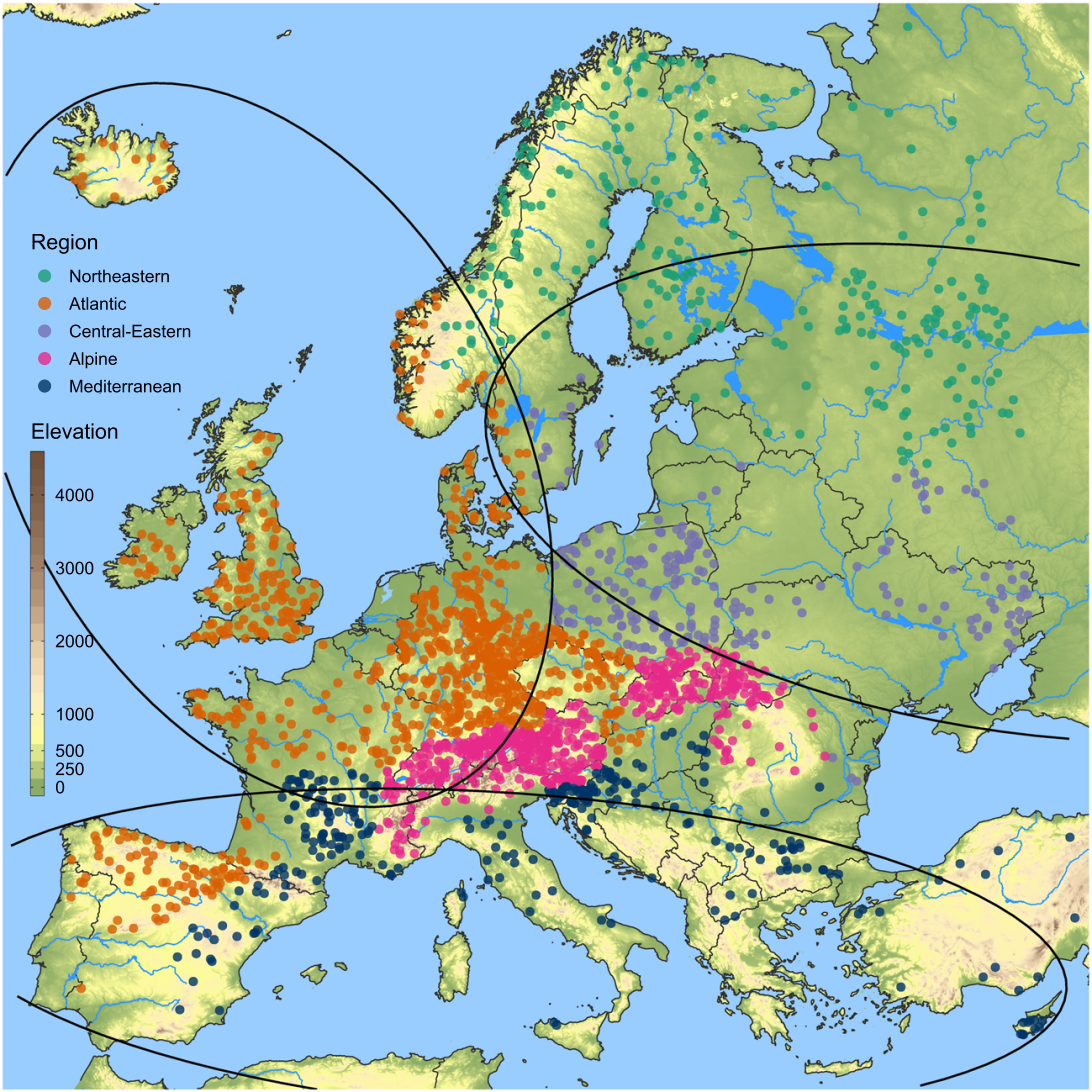
Location of the 2,201 hydrometric stations analyzed. Colors of dots indicate five hydroclimatic regions (northeastern, Atlantic, central eastern, Alpine, and Mediterranean). Ellipses indicate the three hydroclimatic regions (eastern, northwestern, and southern) of Blöschl, Hall, et al. (2019). Background color is elevation.

The selection resulted in 2,201 series with a median catchment area of 376.6 km^2^ and approximately 61% of the series consisting of 51 years. Europe was subdivided in two ways to capture regional patterns of flood‐rich and flood‐poor periods (Figure 2). The first division consists of five regions representing hydroclimatic variability which are a particularization of the eleven biogeographic regions of Roekaerts (2002), guided by the flood seasonalities of Blöschl et al. (2017) and Hall and Blöschl (2018). The second partitioning consists of the three hydroclimatic regions identified by Blöschl, Hall, et al. (2019). These regions are marked as ellipses in Figure 2 and are interpreted as homogeneous regions with respect to change patterns in annual peak discharges, as presented in Blöschl, Hall, et al. (2019).

## 4 Results

### 4.1 Frequency of Flood‐Rich and Flood‐Poor Periods

The results of applying the detection procedure to the 2,201 flood series are summarized in Table 2. As can be seen, the frequency of detected anomalies is above what would be expected from the reference condition of all series being adequately described by an iid‐process. As the individual series have different lengths, the corresponding Type I error probability varies. For instance, with 51 observations and 5‐year thresholds, Table 1 gives *P*(*k* ≥ 6 ∣ *m* = 10,*n* = 51,*a* = 10) = 0.0362 while with 40 observations the probability changes to *P*(*k* ≥ 6 ∣ *m* = 10,*n* = 40,*a* = 8) = 0.0203. Therefore, Table 2 gives the average Type I error probabilities $\bar{\alpha }$ of all series.

**Table 2:** *Number and Frequency of Stations With Detected Flood‐Rich and Flood‐Poor Periods for Thresholds of Different Return Periods*

Anomaly threshold	Number	Frequency (%)	$\bar{\alpha }$ (%)	Frequency/ $\bar{\alpha }$
2‐year flood (flood rich)	179	8.13	2.37	3.43
2‐year flood (flood poor)	279	12.68	4.11	3.09
5‐year flood (flood rich)	210	9.54	3.86	2.47
5‐year flood (flood poor)	200	9.09	4.01	2.27
10‐year flood (flood rich)	120	5.45	1.99	2.74
10‐year flood (flood poor)	220	10.00	4.09	2.44

*Note*. $\bar{\alpha }$ is the average probability of Type I error of all series. About 2 to 4 times more stations with flood‐rich/poor periods are detected than would be expected by chance. The last column gives the ratio of frequency of the stations with detected anomalies and the average significance level $\bar{\alpha }$ to assist in interpretation but is not considered a quantitative result.

Overall, the observed frequencies are about two to four times larger than the average Type I error probability, indicating temporal clustering in the series. The largest frequency of anomalies is detected for 2‐year flood thresholds. The frequency tends to decrease with increasing return period, which is consistent with Merz et al. (2016) and Liu and Zhang (2017) and can be partly attributed to the amount of available data and the applied methodology.

The lowest frequency of observed anomalies (5.45%) is obtained for flood‐rich periods and 10‐year flood thresholds. This can be expected since the corresponding Type I probability is the lowest. Additionally, at most five 10‐year (or larger) events are possible in each series due to the maximum record length of 51 years. The small number of events results in relatively large step changes in the probability distribution of the Scan statistic which reduces the power of the method and thus the frequency of detected anomalies.

Most of the series for which flood‐rich periods are detected contain exactly one flood‐rich period. For example, for 5‐ and 10‐year thresholds, either one or no anomaly is detected in all series. For 2‐year thresholds, two anomalies are detected in five series (which are flood‐poor anomalies). Table 3 gives the frequency of stations for which a flood‐rich period of a given length was detected for each window size. For 2‐ and 10‐year thresholds, the most flood‐rich periods are detected for 15‐year windows, indicating decadal variability. For a 5‐year threshold, the most anomalies are detected for 10‐year windows, but anomalies for 15‐year windows are also common. This is in contrast to the findings of Merz et al. (2016) where the frequency of clusters decreased with the window size. Part of the difference may be due to the difference in type of data (annual versus POT series) and part to the different coverage (Europe vs. Germany).

**Table 3:** *Frequency of Stations With Detected Flood‐Rich Periods for a Given Window Size and a Given Return Period*

	Frequency (%)		
Anomaly threshold	5‐year window size	10‐year window size	15‐year window size
2‐year flood (flood rich)	0.00	4.13	6.32
5‐year flood (flood rich)	1.73	7.91	3.86
10‐year flood (flood rich)	1.27	1.73	3.73

For flood‐poor periods, no multiple window sizes were used and the duration of the anomaly is a result of the analysis. For 2‐, 5‐, and 10‐year thresholds, the average duration of the anomalies was found as 9.3, 22.9, and 33.1 years, respectively. The longer duration of the anomalies for higher thresholds results from the fact that the absence of rare (high return period) events is statistically less unusual than the equally long absence of more common events.

### 4.2 Spatial Patterns of Flood‐Rich and Flood‐Poor Periods

**Figure 3:**
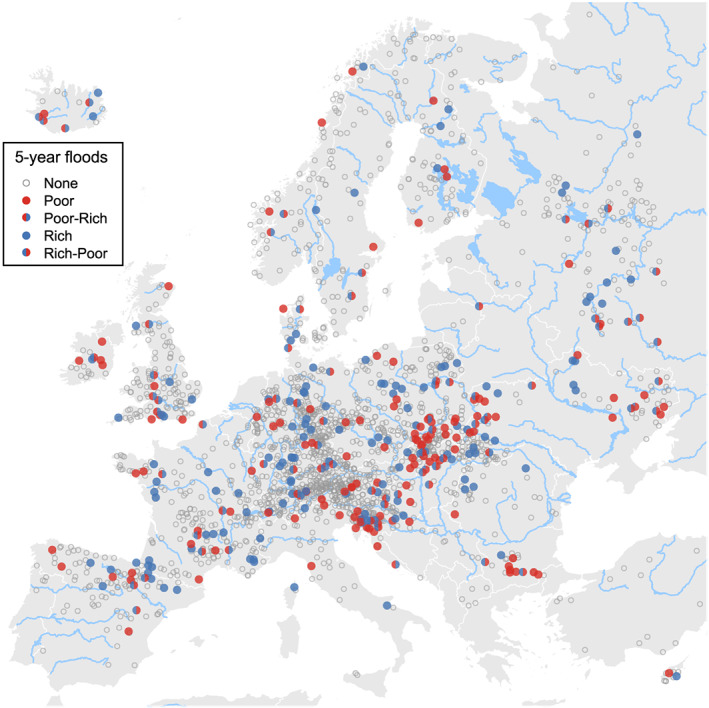
Stations with flood‐rich and flood‐poor periods for a 5‐year flood threshold. Blue indicates stations with flood‐rich periods, and red stations with flood‐poor periods. Rich‐poor refers to stations where first a flood‐rich and then a flood‐poor periods were detected. Poor‐rich refers to analogous combinations. Small gray circles indicate stations without anomalies.

Figure 3 shows the stations with flood‐rich and flood‐poor periods detected during 1960–2010 based on a 5‐year threshold. They are 15.13% of all stations and occur in every region of Europe, with a slight emphasis on the west of the Ukraine and the coast of Croatia and Slovenia. The corresponding percentages for the five regions (northeastern, Atlantic, central eastern, Alpine, and Mediterranean) are 10.42%, 13.19%, 28.37%, 12.75%, and 20.44%, respectively. There is little geographic coherence in Figure 3, which suggests that the exact location of stations with anomalies are less affected by regional climate, which tends to be more homogeneous in space, than by local effects including local climatic variability, geology, vegetation, and/or random elements (see, e.g., Szolgayova et al., 2014 and Rogger et al., 2017 for a discussion of the controls on the temporal clustering of streamflow and land use change effects on floods). Please note that Figure 3 is not a representation of changes in flood‐rich/poor periods but a representation of the total number of flood‐rich/poor periods, which is a reflection of the degree of temporal clustering of floods. On the other hand, the regional differences in the frequencies (e.g., much larger frequency in central eastern than Atlantic) may be more related to differences in the flood generating mechanisms such as a prevalence of snowmelt floods in eastern Europe (Kemter et al., 2020).

**Figure A1:**
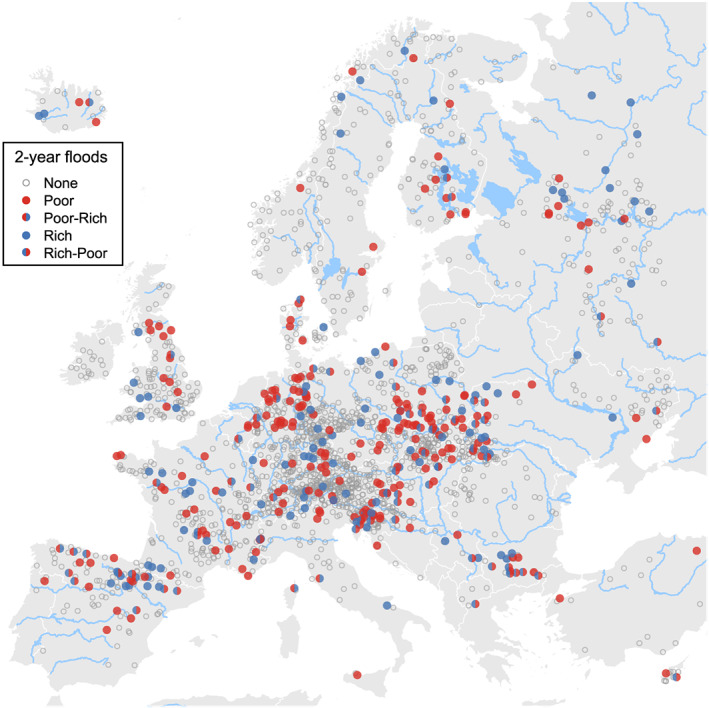
Stations with flood‐rich and flood‐poor periods as in Figure 3 but for a 2‐year flood threshold.

**Figure A2:**
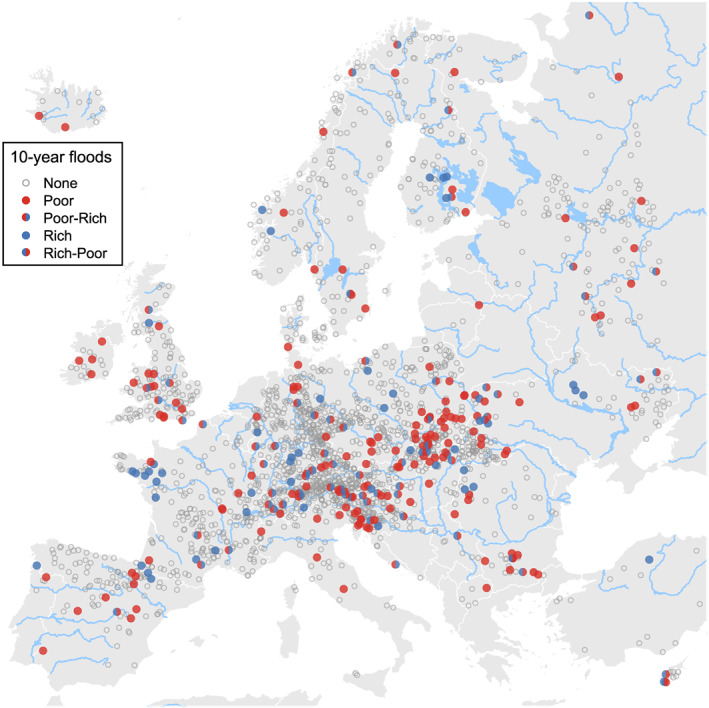
Stations with flood‐rich and flood‐poor periods as in Figure 3 but for a 10‐year flood threshold.

Figures A1 and A2 give the corresponding maps for 2‐ and 10‐year thresholds, and the percentages of the anomalies are 17.54% and 12.31%, respectively. There are relatively few stations that show anomalies for all three thresholds (2.23%, Table 4). Only 9.68% of the series exhibit an anomaly for at least two different return periods, whereas 23.4% of series are assigned an anomaly for only one out of three possible return periods.

**Table 4:** *Number and Frequency of Stations With Detected Flood‐Rich and Flood‐Poor Periods for Different Return Periods in the Entire Data Set (2,201 Stations)*

Anomaly threshold	Number	Frequency (%)
None	1,473	66.92
2‐year flood (only)	244	11.09
5‐year flood (only)	146	6.63
10‐year flood (only)	125	5.68
2‐ and 5‐year floods	67	3.04
2‐ and 10‐year floods	26	1.18
5‐ and 10‐year floods	71	3.23
2‐, 5‐, and 10‐year floods	49	2.23
Total	2,201	100.00

*Note*. Anomalies refer to either a detected flood‐rich or/and a detected flood‐poor period for the respective return period. Lines are mutually exclusive and collectively exhausting.

### 4.3 Regional Temporal Patterns of Flood‐Rich and Flood‐Poor Periods

**Figure 4:**
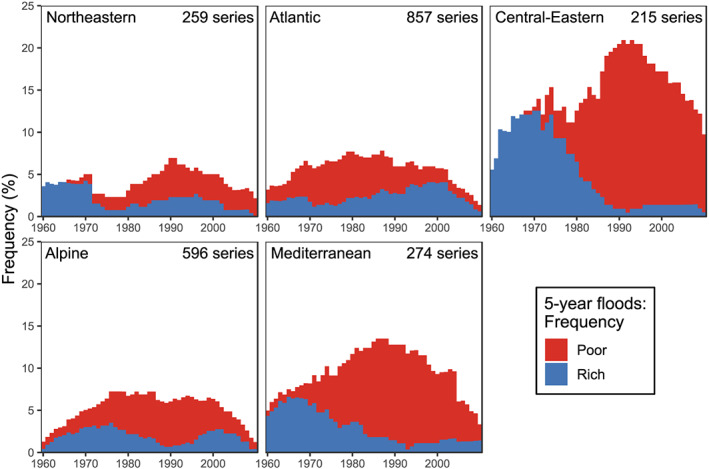
Frequency (%) of stations that exhibit a flood‐rich or a flood‐poor period in a given year for a 5‐year flood threshold in the five geographical regions of Figure 2. Flood‐poor frequencies (red) are stacked on top of flood‐rich frequencies (blue).

In order to provide a temporal assessment of the flood‐rich periods, for a given threshold return period, results for all three windows lengths (5, 10, and 15 years) were combined to obtain composite periods. As the flood‐rich periods of different (and also the same) window lengths sometimes overlap, the duration of these composite flood‐rich periods may differ from the prescribed window lengths. For each year, we then counted the number of stations that were either in a (composite) flood‐rich period, a flood‐poor period or in neither of them. Figure 4 displays the results for a 5‐year threshold for each of the five hydroclimatic regions from Figure 2. In the first decade (1960–1970), the northeastern, central eastern, and Mediterranean regions exhibit almost exclusively flood‐rich periods. There appears to exist a turning point around 1970, when the number of stations with flood‐poor periods starts to increase and those with flood‐rich periods start to decrease. From to 1980 to 2010 the number of stations with flood‐poor periods in these regions is consistently much higher than those with flood‐rich periods. The temporal patterns in these three regions are surprisingly similar and very pronounced, although the absolute frequencies of the anomalies in the central eastern region is substantially higher (frequencies of up to 20%) than in the northeastern (up to 7%) and Mediterranean (up to 12%) regions. In the Atlantic region, flood‐poor periods dominate until around 1993 when the flood‐rich periods take over in terms of the frequency of stations which is consistent with previous studies (e.g., Macdonald & Sangster, 2017). The Alpine region shows a dominance of flood‐rich stations at the beginning of the record, a period of mostly flood‐poor stations in 1975–2000, and again an increase of flood‐rich stations at the end of the record. In most regions, the frequencies at the beginning and/or end of the observation period are smaller than in the middle. At least partly, this is related to the power of the Scan statistic, which is higher if the anomalous segment of the time series lies in the middle of the record (Fu & Curnow, 1990).

**Figure A3:**
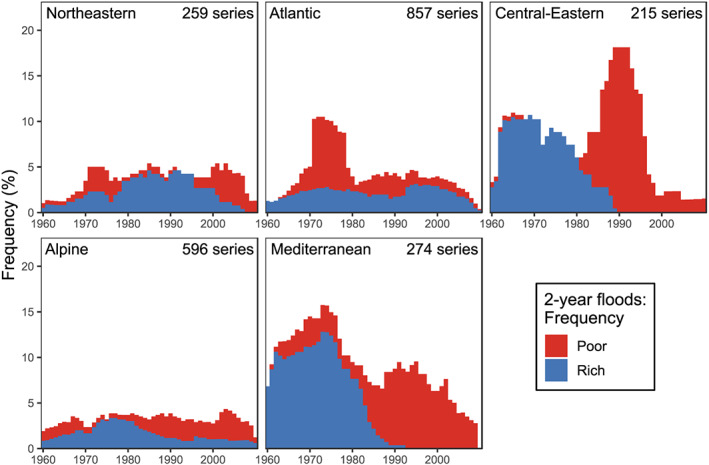
Frequency (%) of stations that exhibit a flood‐rich or a flood‐poor period in a given year as in Figure 4 but for a 2‐year flood threshold.

In the case of 2‐year thresholds (Figure A3), the flood‐rich periods at the beginning of the observational period are even more pronounced for the central eastern and Mediterranean regions from 1960 to 1980, and, surprisingly, there are almost no flood‐rich periods detected at all from 1985 to 2010, indicating a strong signal. In the Atlantic region, a spike of flood‐poor periods arises in the 1970s, which is caused by a group of stations simultaneously exhibiting flood‐rich periods in a small region in Denmark, the northwest of Germany and Belgium. For a 10‐year threshold (Figure A5), flood‐rich periods are less common, as indicated by Table 2, which may be partly due to the choice of the critical number of exceedances, *k*, resulting in low *α*, in combination with the limited length of the time series. This results in big steps in the distribution of the corresponding Scan statistics, which reduces the frequency of the detected periods. The temporal patterns of flood‐rich and flood‐poor periods of the 2‐, 5‐, and 10‐year thresholds are, however, similar. Given that there are relatively few stations that show anomalies for all three thresholds, this may be surprising. The likely reason for the consistency of the time patterns is that it may not be exactly the same station exhibiting an anomaly but a neighboring station, because of a coherent spatial signal and some loss of information for individual stations due to the dichotomization of the data. This finding lends credence to the regional results of the anomalies, suggesting that there is a consistent regional hydrological signal manifesting itself at different stations.

**Figure 5:**
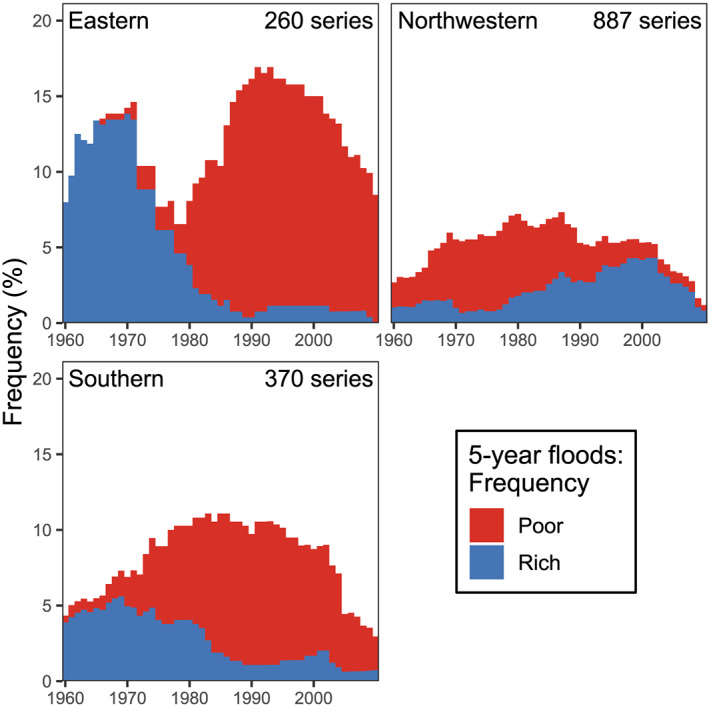
Same as Figure 4 (5‐year flood threshold) but for three hydroclimatic regions (ellipses in Figure 2).

**Figure A4:**
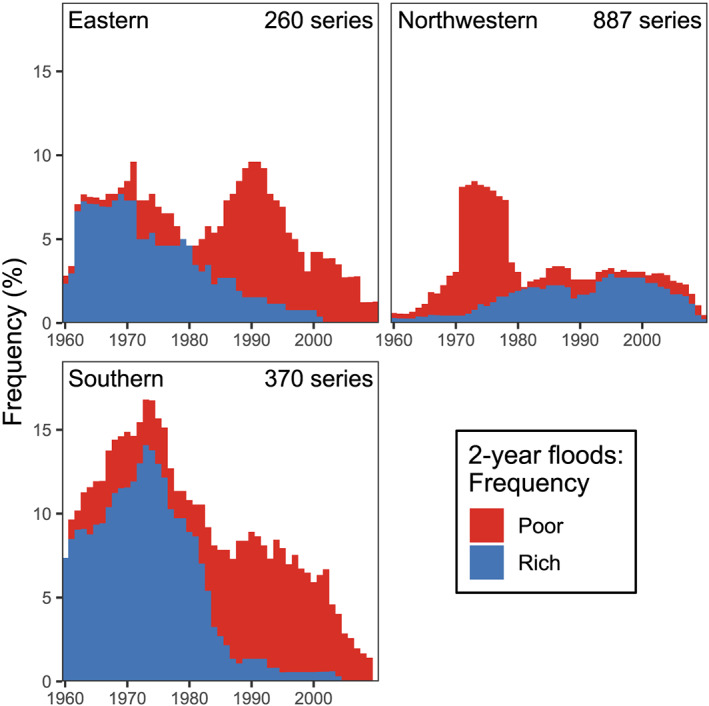
Frequency (%) of stations that exhibit a flood‐rich or a flood‐poor period in a given year as in Figure 5 but for a 2‐year flood threshold.

Figure 5 displays the temporal evolution of the flood‐rich and flood‐poor periods for 5‐year events and the three regions of Blöschl, Hall, et al. (2019). Although the three regions are not fully aligned with the five regions of Figure 4, the temporal patterns are similar. In the eastern and southern regions, flood‐rich periods are dominant from 1960 to 1970, followed by a gradual decrease of flood‐rich periods and an increase of flood‐poor periods until 1980 and, after that, consistently high flood‐poor and low flood‐rich frequencies. The northwestern region in this plot is similar to the Atlantic region in Figure 4 with flood‐poor periods dominating until around 1980 and flood‐rich periods gradually taking over after that. In the case of 2‐year thresholds (Figure A4), the flood‐rich periods in the southern region are more pronounced and their frequency peaks in the first half of the 1970s. For the 10‐year thresholds (Figure A6), flood‐rich periods are, again, less common than for the lower return periods, and flood‐poor periods are more common.

### 4.4 Spatiotemporal Patterns of Flood‐Rich and Flood‐Poor Periods

**Figure 6:**
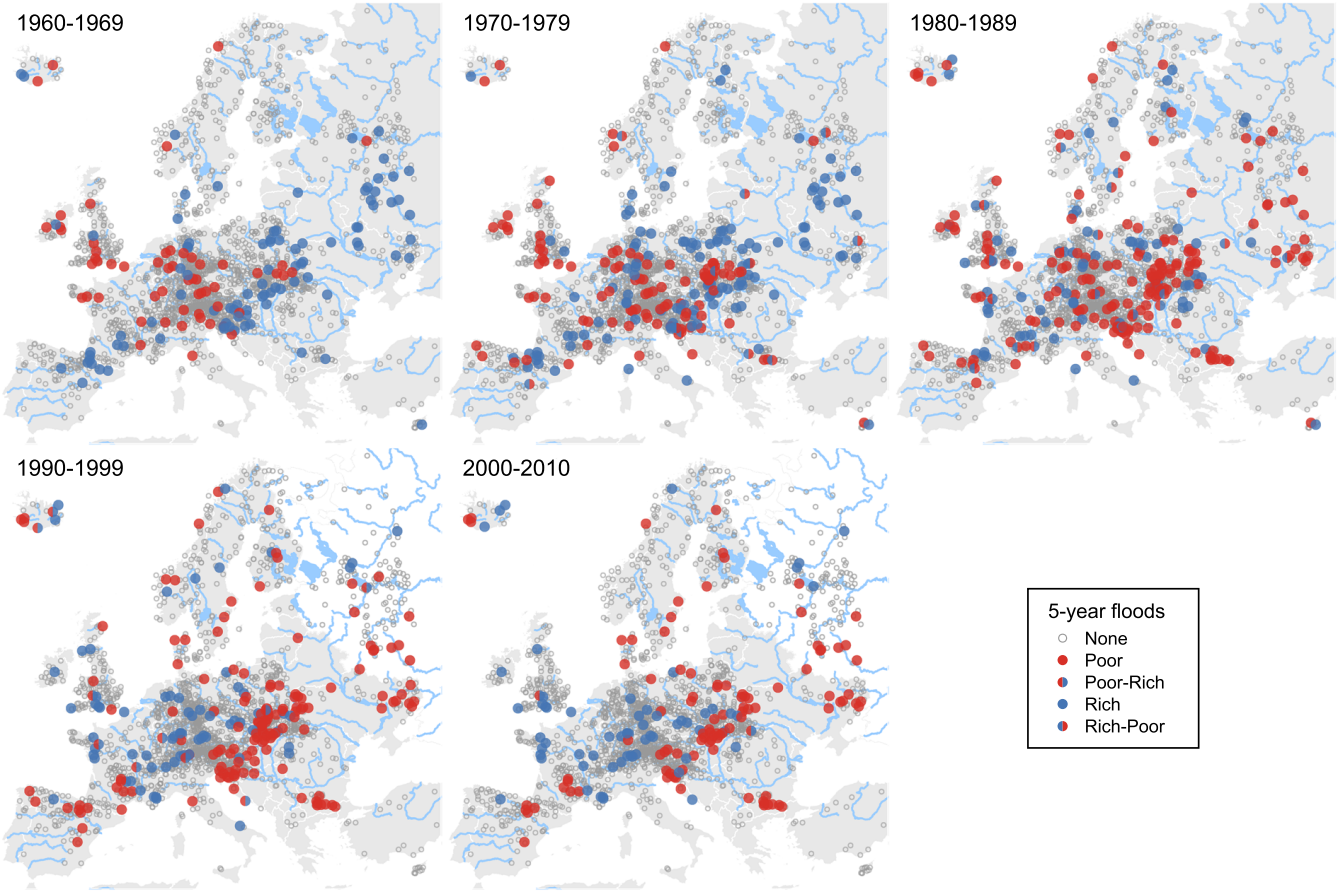
Spatiotemporal results of the detection procedure for flood‐rich and flood‐poor periods for a 5‐year flood threshold. Blue indicates stations with flood‐rich periods, red stations with flood‐poor periods. Rich poor refers to stations where first a flood‐rich and then a flood‐poor periods were detected. Poor rich refers to analogous combinations. Small gray circles indicate stations without anomalies.

Figure 6 depicts the temporal evolution of flood‐rich and flood‐poor periods for 5‐year thresholds in a spatiotemporal context. In the first decade, 1960–1969, flood‐poor periods occur mainly in the Atlantic region including southern England, northern France, and Germany, while the flood‐rich periods mainly occur in eastern Europe. In the following decade, 1970–1979, the patterns are similar. The period 1980–1989 is quite different in the east and the south with the frequency of flood‐poor periods increasing, while the northwest is rather similar to the previous decades. The period 1990–1999 exhibits a clear change in patterns. The region of Atlantic influence starts to exhibit a much larger number of flood‐rich periods, and the east and south an even clearer pattern of flood‐poor stations. In the final decade, 2000–2010, this pattern manifests itself even more clearly. Essentially all the detected anomalies in the region with Atlantic influence are flood rich, while almost all the detected anomalies in the east and south are flood poor. The patterns for 2‐ and 10‐year thresholds are very similar (Figures A7 and A8). The patterns are spatially more coherent than those in Figure 3 because of the stronger climate effect in a spatiotemporal context.

Table 5 shows the frequency of stations with anomalies partitioned into two time segments. If an anomaly encompasses years from both time segments, it is counted both times. In the eastern region, there is a clear and sharp decrease in flood‐rich periods from the first to the second time segment for all return periods, while the flood‐poor periods change relatively less. For the northwestern region, the decrease in flood‐poor periods for a 2‐year threshold and the increase in flood‐rich periods for a 10‐year threshold are especially noticeable. In the southern region, a steep decrease in flood‐rich periods is apparent, especially for the 2‐year threshold. This is consistent with Tramblay et al. (2019), who report a decrease in the frequency of annual flood discharges above high quantiles of daily flows for Mediterranean basins. Overall, the patterns of increases and decreases are consistent across return periods, but there are differences. In fact, while a comparison of the absolute frequencies between return periods needs to be done with care because of potential effects of the method, a comparison of the changes with time between return periods is considered more robust. In the eastern region, the relative changes in the frequencies *f*_*1*_ and *f*_*2*_ of the two time segments, (*f*_*2*_ − *f*_*1*_)/*f*_*1*_, are 72% and 84% (decreases) for the 2‐ and 10‐year thresholds, respectively, suggesting that the change toward fewer floods is slightly more relevant for higher return periods. In the northwestern region, the corresponding figures are 96% and 264% (increases), suggesting that the change toward more frequent floods is much more relevant for higher return periods and, in the southern region, the corresponding figures are 78% and 67%, suggesting that the change toward fewer floods is slightly less relevant for higher return periods.

**Table 5:** *Frequency (%) of Stations With Detected Flood‐Rich and Flood‐Poor Periods for Different Return Periods in the Three Hydroclimatic Regions, Separately for the First and the Second half of the Observation Period (at Least 1 Year of the Anomaly in That Half of the Observation Period)*

	Eastern		Northwestern		Southern	
Anomaly threshold	1960–1984	1985–2010	1960–1984	1985–2010	1960–1984	1985–2010
2‐year flood (flood rich)	9.62	2.69	2.48	4.85	14.59	3.24
2‐year flood (flood poor)	5.77	11.54	8.57	2.14	9.73	14.32
5‐year flood (flood rich)	15.77	2.31	3.72	6.88	8.38	3.78
5‐year flood (flood poor)	9.23	16.15	5.86	4.74	9.19	11.08
10‐year flood (flood rich)	7.31	1.15	1.24	4.51	4.05	1.35
10‐year flood (flood poor)	15.00	15.38	7.10	7.10	11.62	11.62

## 5 Discussion

### 5.1 Do Flood‐Rich and Flood‐Poor Periods Exist?

This study detected about 2 to 3 times more flood‐rich/poor periods (i.e., interannual clustering) in Europe than what would be expected by chance (Table 2). For example, for a return period of 5 years, 9.54% of the stations showed flood‐rich periods. This is similar to the percentage of Merz et al. (2016) (between 6% and 13%, depending on the specification of the method) for Germany. On the other hand, Mediero et al. (2015) found clustering in 24% of the stations (their Table 4 in Europe for the period 1956–1995 and 43% for 1900–1999. The smaller percentages found here may be partly due to the shorter record lengths and a method that gives lower frequencies at the beginning and end of the observation period due to boundary effects (Fu & Curnow, 1990). Liu and Zhang (2017) report clustering in about half of the investigated series in Australia when 2‐year events are considered (their Figure 8). This is higher than the present study, which may be related to the stronger role of decadal climate oscillations in Australia (Shi et al., 2017). While the occurrence of temporal clustering of floods is not fully understood, atmospheric processes, linked to the ocean, are probably most relevant, with geology, soils, and vegetation probably playing lesser roles (Szolgayova et al., 2014). Hofstätter and Blöschl (2019) found that some flood producing cyclones over central Europe tend to cluster as, during the event, the large‐scale atmospheric circulation is changed into a state favoring the development of successive cyclones of a similar type.

### 5.2 Where and When Did the Flood‐Rich and Flood‐Poor Periods Occur?

While Mediero et al. (2015) found more frequent clustering in the Atlantic and continental regions of Europe using 103 flood records, with the more extensive data set of 2,201 series in this paper, this is no longer the case. For the period 1960–2010, the frequency of clustering (or anomalies) is rather uniform in Europe (Figures 3, A1, and A2).

However, when considering the spatiotemporal distributions, very clear patterns emerge. In the East of Europe, a frequency of about 12% of the stations with flood‐rich periods in the 1960s and 1970s decreased to about 1% in the 1990s and 2000s (5‐year return period). In the south, a frequency of about 4–5% decreased to about 1%. However, in the northwest a frequency of about 1% increased to 4–5% (Figure 5). These changes in the number of stations with flood‐rich periods are fully consistent with the flood trends of the mean annual floods evaluated by Blöschl, Hall, et al. (2019). This paper goes beyond the trend analyses of Blöschl, Hall, et al. (2019) by providing more temporal detail on the flood changes.

Using the same data set as in this study, Bertola et al. (2020) investigated regional trends in flood discharges of various return periods in Europe. They report increasing flood discharges in the Atlantic region, where trends are bigger for large return periods and small catchments. They report decreasing flood discharge trends in southern Europe, with a more pronounced decrease for small return periods due to a decrease in soil moisture. For eastern Europe their reported trends are strongly negative for large and small floods and across small and big catchments. These trend findings are consistent with the changes in flood‐rich/poor periods found in this study.

The larger frequencies of the anomalies in central eastern (up to 20%), than in the northeastern (up to 7%) and Mediterranean (up to 12%) regions (Figures 4 and 5) are consistent with the larger negative trends in Blöschl, Hall, et al. (2019) and Bertola et al. (2020) in eastern Europe. This is because of the reduced effect of snowmelt on floods in recent years, which is more relevant in catchments with snowmelt contributions in the central east of Europe than in the north of Europe where temperatures are lower.

Even though the number of individual stations consistently exhibiting anomalies for different return periods is rather low (Table 4), at the regional scale the different return periods do give similar temporal patterns of flood‐rich and flood‐poor periods (e.g., Figures 5, A4, and A6). This finding suggests that the detected anomalies indeed reflect a clear regional signal of changes in the frequency of flood‐rich and flood‐poor periods. A closer examination of the temporal changes of the frequency of stations with flood‐rich periods (Table 5) suggests that the changes toward fewer floods in the east and south do not depend much on the return period, while the change toward more frequent floods in the northwest of Europe is much more relevant for higher return periods. This finding is consistent with studies highlighting the occurrence of heavy rainfall events in northwestern Europe in recent years (e.g., Guo et al., 2019 Otto et al., 2018).

### 5.3 Mechanisms of Flood‐Rich and Flood‐Poor Periods

In order to assess the spatiotemporal patterns of flood‐rich/poor periods a discussion of regional flood generation mechanisms in Europe is in place. Blöschl et al. (2017) analyzed the flood generation mechanism by comparing the temporal evolution of the seasonality of observed floods with those of a number of potential drivers in the past five decades. They found that in northeastern Europe snowmelt was the main driver. In northwestern Europe extreme precipitation played a key role due to the prevalence of shallow soils while in western Europe both sustained winter rainfall and soil moisture were most relevant due to the larger water storage capacity of the soils. Blöschl, Hall, et al. (2019) evaluated the time evolution of maximum annual flood discharges relative to those of the annual maxima of potential drivers with similar findings. In northeastern Europe, snowmelt was identified as the main driver and, in Atlantic western Europe, winter rainfall and soil moisture were important. In central Europe they found a key role of soil moisture in flood generation during spring and summer, and in southern Europe they explained decreasing floods by decreasing soil moisture associated with increasing evaporation. Based on atmospheric reanalysis data, Kemter et al. (2020) conducted a more formal classification of all flood events in the past five decades into potential generating processes. They found snowmelt to be important in the north and east of Europe, soil moisture excess in the Atlantic climate of western Europe, stratiform rainfall in the mountains of central Europe, and rain‐on‐snow in the midmountain ranges of central Europe. Even though the methods and data of the drivers used in these three studies (Blöschl et al., 2017; Blöschl, Hall, et al., 2019; Kemter et al., 2020) were different, the findings regarding flood generation mechanism are consistent.

These mechanisms assist in interpreting the changes in flood‐rich/poor periods obtained in this paper. In eastern Europe, where snowmelt is the main driver of floods, the frequency of snowmelt dominated floods relative to other flood types has decreased during 1960–2010 (Kemter et al., 2020, their Figure 3b) as a result of increasing air temperatures. These processes explain the shift from a high frequency of flood‐rich periods to a high frequency of flood‐poor periods during the study period found here. These changes are not continuous during the observation period, however, but there appears to be little change in flood‐poor periods until the 1970s and a stark increase after (Figures 4 and 5). This nonlinear change is fully aligned with the temporal patterns of air temperatures, which were approximately constant from 1960 to the mid‐1970s with a stark increase after (see, e.g., Figure 2fc of Blöschl, Hall, et al., 2019). The plateau in air temperature until the mid‐1970s is usually interpreted as resulting from the cooling effects of aerosols and orbital forcing (Undorf et al., 2018).

In northwestern Europe, where floods tend to occur in winter, both winter rainfall and soil moisture have increased during 1960–2010 (Myhre et al., 2019 Wilby et al., 2008 Zolina et al., 2010). Specifically, Extended Data Figures 5 and 7 of Blöschl, Hall, et al. (2019) show an increase of both variables during the study period. Given that extreme winter precipitation and soil moisture are the main drivers of floods in this region, the observed changes in these variables explain the shift from a high frequency of flood‐poor periods to a high frequency of flood‐rich periods found here. Consistent with these changes, the relative frequency of soil moisture excess runoff generation relative to other flood types has increased (Kemter et al., 2020, their Figure 3b). Again, these changes are not continuous during the observation period with precipitation particularly increasing since the 1970s (see, e.g., Figure 2abc of Blöschl, Hall, et al., 2019). The shift in extreme precipitation in the past decades is not fully understood but it appears that it is associated with northward shifts of the subpolar jet and corresponding storm tracks since the 1970s, which in turn are associated with more prevalent positive phases of the Northern Atlantic Oscillation (NAO) index and polar warming (Intergovernmental Panel on Climate Change, 2013). There has indeed been a clear upward step in the winter NAO index in the 1970s (Hurrell et al., 2003 Hurrell & Deser, 2009), as demonstrated, for example, by the statistical analysis of Gómez‐Martínez et al. (2018) (see their Figure 7). The storm track analyses of Hofstätter and Blöschl (2019) suggest that cyclones in Europe are more generally synchronized with the NAO and the Arctic Oscillation. These shifts in precipitation then impact on flood occurrence. Figures 4 and 5 (and the associated figures in the appendix) indicate that, in northwestern Europe, the frequency of stations with flood‐rich periods has been increasing since around 1980. The alignment between flood occurrence and NAO is consistent with other studies. Steirou et al. (2019) report a strong influence of the NAO on floods in northwestern Europe, especially in winter, and Zanardo et al. (2019) show a strong correlation between the NAO and flood losses.

In southern Europe, where floods tend to occur in winter, evaporation has increased in the past 50 years as a result of increasing air temperatures which, together with decreasing precipitation, have resulted in a stark decrease in soil moisture (see, e.g., Extended Data Figure 7 of Blöschl, Hall, et al., 2019). Since soil moisture is an important flood driver in this region flood discharges have decreased (Mediero et al., 2014 Tramblay et al., 2019). Additionally, decreasing precipitation (see, e.g., Extended Data Figure 5 of Blöschl, Hall, et al., 2019) has likely contributed to these changes, explaining the shift from a high frequency of flood‐rich periods to a high frequency of flood‐poor periods found here. Again, these changes are not continuous during the observation period, and Figures 4 and 5 suggest that the frequency of flood‐poor periods has been particularly increasing since the 1970s. On the one hand, evaporation may have increased more strongly since the 1970s due to the aerosol driven hiatus in warming before that time (Undorf et al., 2018). On the other hand, this change seems to be related to precipitation changes associated with the northward shifts of the subpolar jet reflected in the upward step of the winter NAO index in the 1970s (Gómez‐Martínez et al., 2018 Hurrell et al., 2003 Hurrell & Deser, 2009). While positive NAO indices are related to above average rainfall and flooding in northwestern Europe, the opposite is the case in southern Europe. For example, Gómez‐Martínez et al. (2018) demonstrated a high inverse correlation between winter NAO and winter precipitation in Spain, which has translated into lower discharges since the late 1970s. As a note of caution, the high frequency of flood‐poor periods in the past few decades in southern Europe needs to be interpreted in the context of the catchment size of the flood data base used here. Small catchments of a few square kilometers are not contained in the data set (the median catchment size is 377 km^2^). As a consequence, since convective floods mainly occur in small catchments (Viglione et al., 2016), the frequency of convective floods relative to other flood types is very low, as demonstrated by the flood classification of Kemter et al. (2020). Since convective storms are expected to increase in a warmer climate (Ban et al., 2015), it is actually possible that, in small catchments in southern Europe, floods have indeed been increasing in the past decades with a prevalence of flood‐rich periods.

### 5.4 Remarks on the Methodology

We propose a method from Scan statistics for identifying flood‐rich and flood‐poor periods in flood discharge records. For the case of flood‐rich periods, we use multiple window lengths in order to generalize the identification beyond the choice of a single window length. For the case of flood‐poor periods, there is no need for multiple window lengths as the scanning for flood‐poor periods can be interpreted as looking for the longest success run in a series, where successes are interpreted as nonexceedances. As soon as the run is unusually long, the respective window size is adopted.

The resulting frequencies of flood‐rich/poor periods are plausible and consistent with trend analyses. However, care must be taken in interpreting the dependency of the frequencies with return period. There is a tendency toward fewer flood‐rich periods with increasing return period. While Merz et al. (2016) also suggested that the frequency of clustering decreases with increasing threshold return period, this finding may be related to the method and amount of available data. For high return periods, the small number of events results in relatively large step changes in the probability distribution of the Scan statistic, which reduces the power of the test. Longer flood records would permit the investigation of clustering of threshold events with higher return periods, which may reveal clustering patterns that are hidden for now.

**Figure A5:**
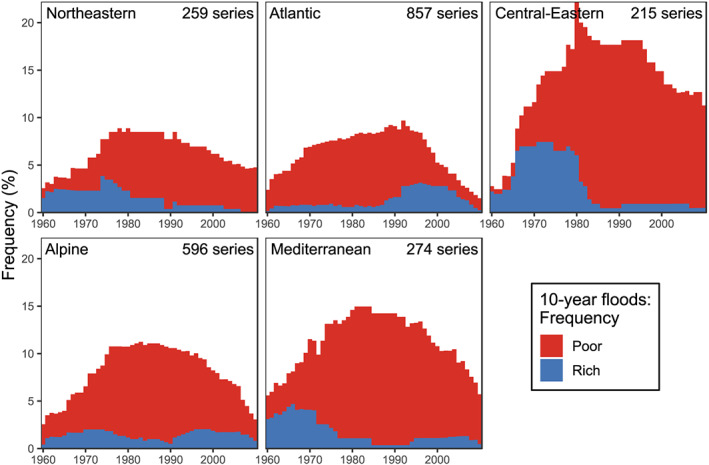
Frequency (%) of stations that exhibit a flood‐rich or a flood‐poor period in a given year as in Figure 4 but for a 10‐year flood threshold.

**Figure A6:**
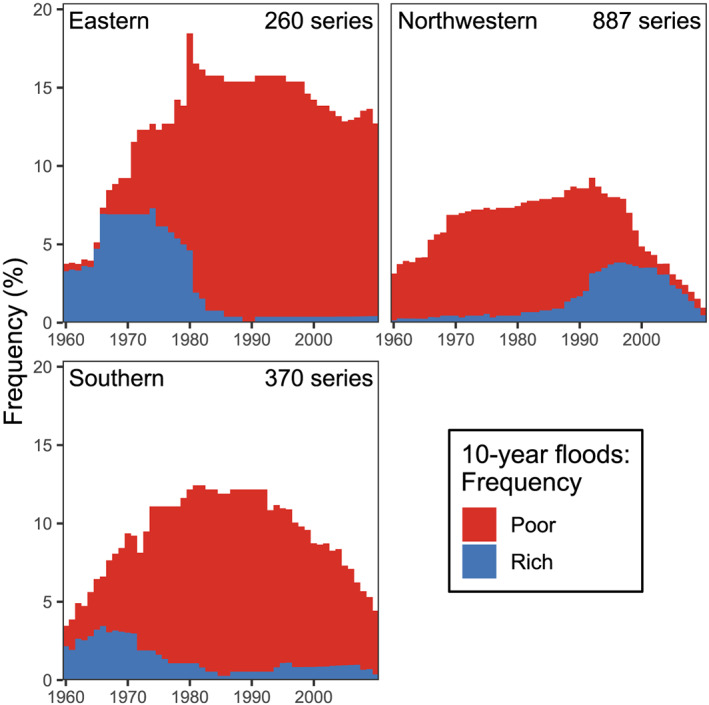
Frequency (%) of stations that exhibit a flood‐rich or a flood‐poor period in a given year as in Figure 5 but for a 10‐year flood threshold.

**Figure A7:**
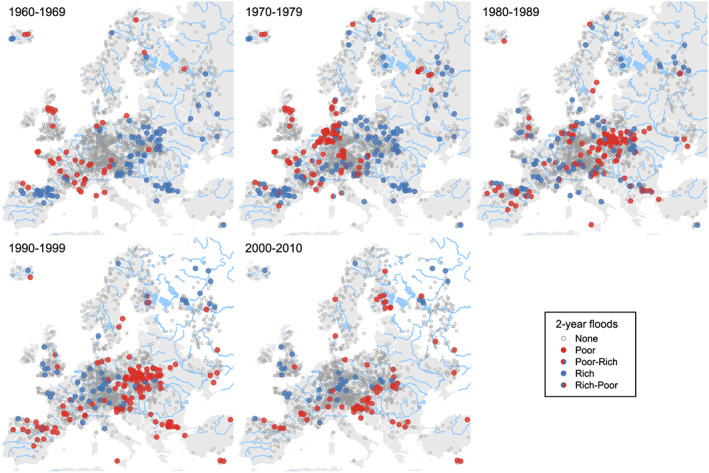
Spatiotemporal results of detection procedure for flood‐rich and flood‐poor periods as in Figure 6 but for a 2‐year flood threshold.

**Figure A8:**
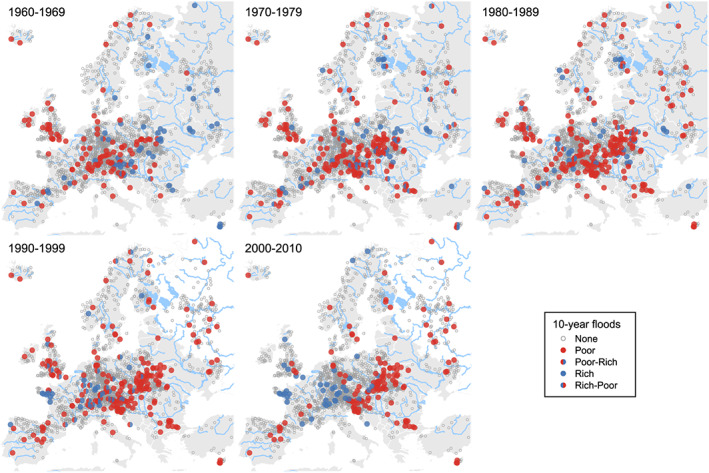
Spatiotemporal results of detection procedure for flood‐rich and flood‐poor periods as in Figure 6 but for a 10‐year flood threshold.

The dichotomization of the flood data by exceedances of a return period threshold allows the application of classical Scan statistics, but it involves some loss of information. Locally, this loss of information may be relevant, but the consistency of the temporal patterns of the frequency of anomalies between different return period thresholds for a given region (e.g., Figures 4, A3, and A5) suggests that this is probably less relevant regionally, which is the focus of the current study. It may not be exactly the same station exhibiting an anomaly at different return period thresholds but a neighboring station due to spatial correlations in the flood data. On the other hand, spatial correlations were not explicitly accounted for in the method. The occurrence of more floods than expected within any given space‐time window may be less surprising when spatial correlations are considered (Serinaldi & Kilsby, 2018). Analyses of the flood discharge records (not shown here) suggest that there is some spatial correlation. The effect of correlations on the results should be considered in future work.

Other potential extensions relate to the nature of the data. The advantage of the proposed approach over that of Merz et al. (2016) and others is that it can be used for both annual and POT series, and there is no need for bootstrapping, as probabilities can be derived exactly, at least for some cases. Also, it can be generalized to higher dimensions (Kulldorff, 1997). Future work may address these generalizations.

## 6 Conclusions

The conclusions of this paper can be summarized as follows.

1. We propose a method from Scan statistics for identifying flood‐rich and flood‐poor periods (i.e., anomalies) in flood discharge records. The method goes beyond trend analysis by providing more temporal detail on flood probability changes.
2. The method is applied to AM flood series but it is also suitable for POT series and can be generalized to higher dimensions, such as space or space‐time.
3. Analysis of 2,201 series of AM peak discharges in Europe between 1960 and 2010 suggests that there is evidence for the existence of flood‐rich and flood‐poor periods that are inconsistent with the assumption of independent and identically distributed random variables. About 2 to 3 times more anomalies were detected than what would be expected by chance.
4. The frequency of the anomalies tends to decrease with an increasing threshold return period, which is consistent with previous studies, but this may be partly related to the method and the record length of only 50 years.
5. Overall, the frequency of anomalies in the observation period does not vary much within Europe.
6. There are, however, clear space‐time patterns of the anomalies. In the northwest of Europe, the frequency of stations with flood‐rich periods tends to increase over time and the frequency of stations with flood‐poor periods tends to decrease over time. In the east and south of Europe, the opposite is the case.
7. There appears to exist a turning point around 1970 (a little later in northwestern Europe) when the frequencies of anomalies start to change most clearly. This turning point occurs at the same time as a turning point of the North Atlantic Oscillation index.
8. In the east and south of Europe the changes toward fewer floods do not depend much on the return period, while in the northwest the change toward more frequent floods is more relevant for higher return periods.

## Appendix: Figures 1

The appendix comprises figures with results analogous to the main text, but for different flood thresholds. Figures A1 and A2 depict stations with detected anomalies for 2‐ and 10‐year flood thresholds. Figures A3, A4, A5 and A6 depict regional temporal patterns of flood‐rich and flood‐poor periods for 2‐ and 10‐year flood thresholds. Figures A7 and A8 depict spatiotemporal patterns of flood‐rich and flood‐poor periods for 2‐ and 10‐year flood thresholds.
